# Relationship between Mood Change, Odour and Its Physiological Effects in Humans While Inhaling the Fragrances of Essential Oils as well as Linalool and Its Enantiomers

**DOI:** 10.3390/molecules18033312

**Published:** 2013-03-13

**Authors:** Yoshiaki Sugawara, Asami Shigetho, Mai Yoneda, Tomoko Tuchiya, Tomomi Matumura, Miki Hirano

**Affiliations:** Department of Health Science, Prefectural University of Hiroshima, Hiroshima 734-8558, Japan

**Keywords:** sensory evaluation, perceived odor quality, fragrance of essential oils, linalool and its enantiomers, fingertip skin temperature change

## Abstract

Humans can detect and discriminate a vast number of odours. The number perceived as distinguishable is estimated to be more than ten thousand. Humans are capable of distinguishing even slight alterations in the structure of an odorous molecule. A pair of enantiomers of an odorant, which possess the same molecular structures except for the chiral position, can trigger profoundly different odour perceptions. How precisely can humans and their olfactory system detect and discriminate such a great variety of odours and such subtle differences in the molecular structures? In a series of studies, we have attempted to examine the relationship between mood change, odour and its physiological effects, by focusing on the possible verbal and non-verbal changes in humans induced by smelling the fragrances of essential oils as well as linalool and its enantiometric isomers. In this article, we provide an overview of our recent verbal and non-verbal studies. We then discuss how our findings may contribute to the assessment of psychophysiological responses of essential oils as well as how our research can contribute to the study of human chemoreception science, by shedding light on the sophistication of the olfactory system in its ability to detect and discriminate odors.

## 1. Introduction

Humans can detect and discriminate a vast number of odours. The number perceived to be distinguishably different is estimated to range from a few thousand to more than ten thousand [1,2]. Humans are capable of distinguishing even slight alterations in the structure of an odour molecule, such as the difference between a pair of enantiomers, which possess the same molecular structure except for the chiral position. Such small molecular differences can lead to profound changes in the perceived odour quality. For instance, with the use of highly purified compounds by gas-liquid chromatography (GLC), it has been shown that (+)-carvone is characterized as a caraway-like scent, while (−)-carvone as a spearmint-like herbal odour [3,4]. Enantiomers of carvotanacetone and *trans*-dehydrocarvone, both synthesized from (+)-carvone, are caraway-like, whereas those prepared from (−)-carvone are spearmint-like [5]. Similarly, (+)- and (−)-linalool are petitgrain-like and lavender-like, respectively [6]. How precisely can humans and their olfactory system detect and discriminate such a great variety of odours and such subtle differences in the molecular structures?

A number of proposals and theories have been presented to explain this issue, with a variety of views involving vibrational energy levels, intermolecular interactions, and molecular size and shape [1]. However, no physiological theories have followed from these ideas. In 1991, a breakthrough was made by Buck and Axel, who identified the genes coding for olfactory receptors [7]. Since this initial discovery in rat, it has been established that the detection of odorants is mediated by a large number of G protein-coupled odorant receptors (Ors) encoded by a multigene family [8,9]. The number of Ors is estimated at ~1,000 in mouse and rat, ~500–750 in human and ~100 in fish [10]. In mammals, Ors are found in the hair-like cilia of olfactory neurons, which are located in the olfactory epithelium at the back of the nose. Thus, initial odour detection is mediated by a vast number of different odorant receptors, located at the entrance of the olfactory system in direct contact with the air, and signals of smell are transmitted to the brain. Under the assumption that the mammalian DNA contains around 100,000 genes, 1% or more of the genome is considered to be devoted to the detection of odours, making the Ors family the largest gene family thus far identified in mammals [11].

The limbic system, which is related to mood, memory, and sexual activity, is closely associated with the olfactory system and is likely to play a principal role not only in discrimination of a great variety of odours and subtle differences in the molecular structures but also in the diverse odour reaction depending on internal and extraneous conditions of the subjects while inhaling fragrant ingredients. Many reports have been published in the literature concerning odours being used to alter mood, alertness, and sexual arousal [12,13,14]. Perfumes, room fragrancers and incense have been used for self-adornment and modification of the living environment from ancient times. The practice of treatment known as Aromatherapy, which began in France in the early 20th century, has been developing rapidly up to the present day. Essential oils are now extensively utilized in the context of Aromatherapy and Aroma Wellness. The following questions arise: How does the brain prompt the range of emotional or behavioral responses that aromas often provoke? To what extent is a particular behaviour or mood governed by the perception of odours?

In our research, we have examined the relationship between mood change, odour and its physiological effects, by focusing on changes in possible verbal and non-verbal responses in humans induced by smelling a fragrant compound [15,16]. In our experimental design, verbal tests always antedated tests of non-verbal functions, because the verbal test was used as a screening test of the essential oils used for non-verbal investigation. To evaluate the perception a given aroma, a sensory test was employed in which the perception of fragrance was assessed by 13 contrasting pairs of adjectives, and the statistical significance of any changes in descriptors was examined by Sudent’s *t*-test. These tests have been carried out for the past decade and the sensory features of 21 essential oils and one monoterpenoid (linalool) as well as its enantiometric isomers were settled so far. Among these, the peppermint sensory evaluation spectrum (in which the mean of the impressions while inhaling the fragrance of peppermint was plotted against the descriptors) was shown to be task dependent. When using an auditory task involving listening to environmental (natural) sounds, the peppermint spectrum was found to be the reverse of the spectrum recorded when undertaking a mental arithmetic task. Similar task dependent effects were found for spearmint and linalool. These findings imply that the perceived odour quality depends on which behavioural task is assigned to the subjects, if the same fragrance given to the subjects. Similarly, same task-dependence of the sensory evaluation spectrum was found for (*RS*)-(±)-linalool. Moreover, the (*R*)-(–)- and (*S*)-(+)-forms of linalool resulted in different sensory spectra when subjects were undertaking mental arithmetic, but gave identical spectra when the auditory task was used. Based on these findings, it can be speculated that enantiomers of linalool may evoke different odour perceptions not only based on chirality but also based on behavioural tasks.

On the basis of these verbal responses in the background, we then studied temperature changes induced by smelling odorants as an indication of changes in non-verbal responses in participants. As a first step, essential oils of lemon, peppermint, spearmint and ylang ylang and linalool served as target odours. Effect of ylang ylang seemed to be relaxing/sedating according to the obtained sensory spectrum and skin temperature changes when the subjects were undertaking the auditory task. However, when the subjects were undertaking mental arithmetic, the effect of ylang ylang was instead in good agreement with “harmonization” (rather than relaxing/sedating), which was introduced by Hongratanaworakit and Buchbauer [17]. Furthermore, when ylang-ylang’s sensory evaluation spectrum and effect on temperature were used as a reference to find odorants with similar properties, peppermint, spearmint and linalool were also categorized as “harmonizing”. In contrast, lemon had a task-dependent dual effect on skin temperature (a decrease during mental arithmetic and an increase during the auditory task), indicating that the task-dependence of verbal and non-verbal responses was determined by the nature of the tested odours.

In this article, we will discuss this task-dependence of psychophysiological responses to odours in the context of how research on this phenomenon might contribute not only to the assessment of psychological and physiological effects of essential oils, but also to the study of human chemoreception by shedding light on the sophistication of the olfactory system. Although sensory perception and physiological endpoints are rarely recorded in the same study, this approach has the potential to be highly informative.

## 2. Sensory Evaluation of Perceived Odour Quality of Essential Oils and Linalool and Its Enantiometric Isomers

Sensory evaluation is a verbal (semantic) method of measuring consciousness developed primarily by experimental psychology and mathematical psychology [18,19,20,21,22]. In an attempt to shed light on the possible psychological effects inhaling essential oils on humans, we have developed a method for the quantification and statistical analysis of the sensory perception of odours [15,16,23,24,25,26,27,28,29,30]. In this section, we deal first semantic (sensory) evaluation of the perceived odour (aroma) quality in humans while inhaling the fragrances of essential oils as well as linalool and its enantiometric isomers.

Essential oils were purchased from Fleur (London, UK) and linalool was purchased from Kanto Kagaku Co., Ltd. (Tokyo, Japan). To identify the optimal concentration of each odorant for inhalation experiments, preliminary sensory tests were performed according to the method of Sugawara *et al.* [25]. Serially diluted solutions (1/1, 1/5, 1/10, 1/50, 1/100, 1/1,000 and 1/10,000) of the odorants in deodorized diethyl phthalate were presented to several judges (usually five) via an inhalator composed of a glass inhalator device and a 300 mL flask with a ground-glass stopper. The inhalator flask was loaded by applying 200 μL of diluted odorant to a small strip of filter paper placed at the bottom of the flask. The flask was sealed with a ground-glass stopper and moistened with odorant. The optimal concentration of optically active linalools will be detailed later.

The following applicable odour detection threshold scores were used: 0, odourless; 1, odour barely detectable and the nature of the odour cannot be ascertained; 2, very weak odour but the nature of the odour can be discriminated; 3, weak odour but the odour can be readily detected; 4, strong odour; and 5, odour so strong that it cannot be tolerated. The five judges were requested to score each test solution from 0 to 5. Concentrations receiving a score of 3 or above were used for experiments. For example, we used dilutions of 1/5 for lemon, 1/10 for spearmint, ylang ylang and linalool, and 1/100 for peppermint.

Aroma perception was evaluated by the following 13 descriptors, consisting of contrasting pairs of adjectives: fresh–stale, soothing–activating, airy–heavy, plain–rich, natural–unnatural, elegant–unrefined, soft–strong, pleasant–unpleasant, warm–cool, comfortable–uncomfortable, woodsy–not woodsy, floral–peppery, lively–dull. We used the Uchida-Kraepelin test as a mental arithmetic task, listening to environmental (natural) sounds as an auditory task, and stepping up and down a step as a physical task. The Uchida-Kraepelin test involves administering adjacent rows of numbers (100 numbers per row) to the subjects. Subjects perform simple additions using numbers within a row. Subjects worked on each row for 40 s before changing to the next row (5 min total). The auditory task (5 min total) consisted of sitting on a chair and listening to natural sounds such as bird song and the murmuring of a small stream on a compact disc player. The physical task involved stepping up and down a 20 cm step at a rate of 30 times per min for 5 min.

The sensory perception test was conducted before and after the task. The 13 descriptors were scored on an 11-point scale (–5 to +5), with 0 as the middle score and without any symbolic representation of the numbers, similar to the Likert scale [31]. The differences between pre- and post-task scores (post minus pre) for each of the descriptors were evaluated by *t*-tests. The mean difference in the score of each descriptor was plotted against the 13 descriptors (sensory evaluation spectrum). The statistical significance of each descriptor was marked and scored as follows: * (asterisk) and significance score 1 if the difference was significant with *p* < 0.05; ± and significance score 0.5 if the difference was significant with *p* = 0.05–0.1; and unmarked and significance score 0 if the difference was insignificant with *p* ≥ 0.1. The sum of these scores provided the following total significance score = Σ^13^_i = 1_ (significance score of descriptor)_i_. We used this total significance score as an index of whether or not the sensory profile could be regarded as statistically significant as a whole [16]. We applied a sign test with *n* = 13, since 13 pairs of descriptors were used in our sensory test. Based on this, sensory spectra could reach significance (*p* < 0.05) as a whole if the number of descriptors regarded as significant (*p* < 0.05) by the *t*-test was >10 out of the 13 descriptors, whereas a value of <3 means that the null hypothesis can be rejected.

One thousand six hundred and thirty-one subjects formed the sensory test panel and completed the study. Among these, 90 were male students from Hiroshima University aged between 18 and 24 years. The remaining subjects were female students at Hiroshima University, Prefectural University of Hiroshima (formerly Hiroshima Prefectural Women's University, but renamed as of April 1, 2005) or Suzugamine Women’s College, with ages ranging from 18 to 22 years. So, the proportion of males was only about 5%. No participant overlapped as a panellist.

As summarized in Table 1, these types of tests have been carried out for the past decade, and the sensory features of the following 21 essential oils and one monoterpenoid (linalool) as well as its enantiometric isomers have been detailed so far: basil (*Ocimum basilicum*), bergamot (*Citrus bergamia*), cardamom (*Elettaria cardamomum*), chamomile (*Matricaria chamomilla*), cinnamon (*Cinnamomum zevlanicum*), clove (*Syzigium aromaticum*), cypress (*Cupressus sempervirens*), geranium (*Pelargonium graveolens*), ho leaf/wood (*Cinnamomum camphora*), juniper (*Juniperus communis*), lavender (*Lavandula angustifolia*), lemon (*Citrus limon*), lime (*Citrus latifolia*), marjoram (*Origanum majorana*), orange (*citrus sinensis*), palmarosa (*Cymbopogen martini*), peppermint (*Mentha piperita*), rosemary (*Rosmarinus officinalis*), sandalwood (*Santalum album*), spearmint (*Mentha spicata*) and ylang ylang (*Cananga odorata*).

## 3. Sensory Profiles of Peppermint and Spearmint Essential Oils and Linalool as a Function of Behavioural Task (Extraneous Condition Assigned to the Subjects)

**Figure 1:**
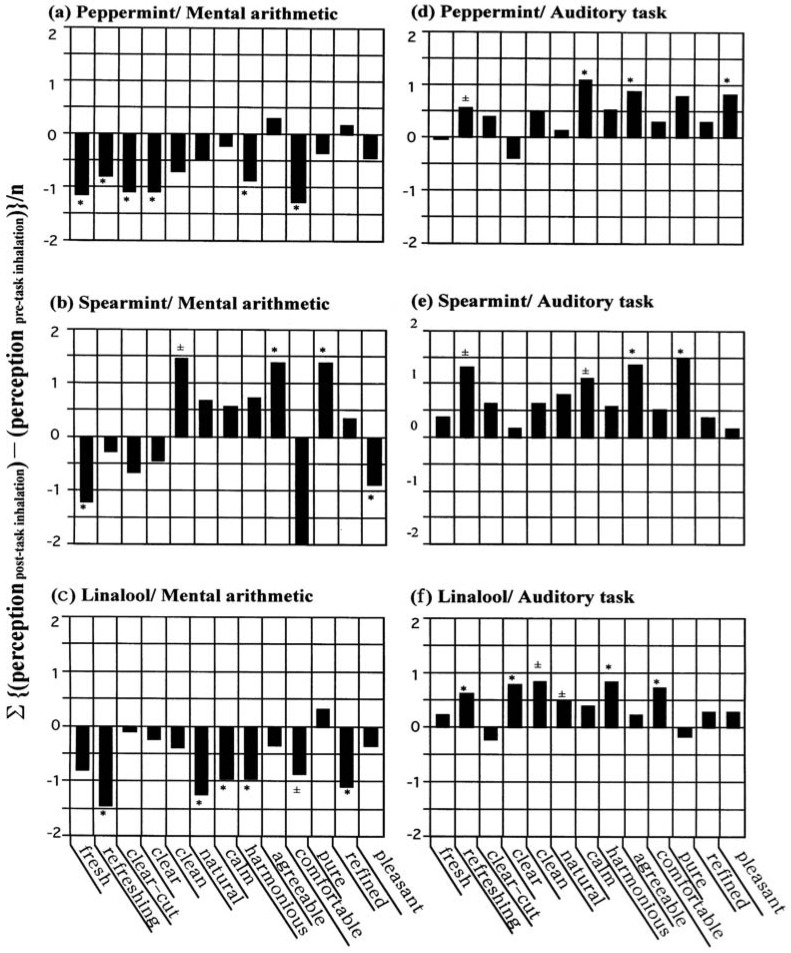
Sensory spectra of peppermint and spearmint essential oils and linalool as a function of behavioural task. Redrawn from Sugawara *et al.* [15]. A sensory test was conducted twice before and after the task assigned to the subjects, in which aroma perception was evaluated by 13 impression descriptors consisting of contrasting pairs of adjectives. The pre-post task difference in the score of each of the impression descriptors is plotted on the ordinate as a bar graph. The statistical significance evaluated by *t*-test of each descriptor was marked with a single asterisk (*) if the pre-post impression difference was regarded significant with *p* < 0.05, ± if regarded significant with *p* = 0.05–0.1, and unmarked if *p* ≥ 0.1. The number of subjects was (**a**) 20, (**b**) 18, (**c**) 20, (**d**) 23, (**e**) 18 and (**f**) 22.

As a measure (sensory profiling) of the perceived odor quality in participants after inhalation of a given aroma on the basis of the task-dependent sensory questionnaire assessments, we employed a sensory evaluation spectrum. It is a bar graph in which the mean of the pre-post task difference between the score of post-task inquiry and pre-task inquiry (post minus pre) was plotted against the 13 impression descriptors. Typical examples are shown in Figure 1. In each spectrograph, if the descriptors regarded as significant by *t*-test have a positive value and are shown above the horizontal axis, this means a positive (or favorable) correlation between the fragrance of a given aroma and the type of task from the viewpoints of “fresh,” “airy,” “elegant,” “pleasant,” “comfortable,” and the other setting descriptors; if negative values appear below the axis, this suggests an unfavorable (negative) correlation between the fragrance and the type of task in terms of “stale,” “heavy,” “unrefined,” “unpleasant,” “uncomfortable,” and the other setting descriptors.

In the case of peppermint spectrum in association with mental arithmetic, as shown in Figure 1a, there was an unfavorable (or negative) correlation between the fragrance and the type of task assigned to the subject. On the other hand, peppermint spectrum in relation to the auditory task (Figure 1d) was shown to be positive correlation between the fragrance and the task. Similar results were found for spearmint (Figure 1b,e) and linalool (Figure 1c,f). This implies that different behavioural tasks influence the perceived odour quality of essential oils. Moreover, the finer nuances visible in the appearance of each spectrum show how this method of sensory profiling can be practical for assessing odour perception in participants.

**Table 1:** Summary of the total significance scores (see text) resulting from statistical analysis on verbal (semantic) responses to odorant of the following 21 essential oils and linalool as well as its enantiometric isomers.

The obtained total significance score for a given aroma in relation to the task assigned to the subjects with the number of subjects in parenthesis			
Odorant	Task assigned to the subjects		
	Mental arithmetic	Auditory task	Physical task
**(1) essential oils**			
basil	5.0 ( *n* = 22)	2.0 ( *n* = 22)	1.0 ( *n* = 21)
bergamot	2.0 (24)	2.5 (18)	4.0 (22)
cardamon	0.0 (24)	3.5 (23)	0.5 (21)
chamomile	0.5 (21)	1.0 (23)	1.5 (17)
cinnamon	2.0 (36)	8.5 (41)	1.0 (23)
clove	0.5 (20)	2.0 (19)	0.0 (17)
cypress	0.5 (20)	1.0 (18)	3.5 (24)
geranium	5.5 (21)	2.5 (30)	1.5 (18)
ho leaf/wood	1.0 (19)	2.5 (18)	1.5 (23)
juniper	6.5 (17)	6.5 (19)	1.5 (19)
lavender	0.0 (20)	1.5 (21)	1.0 (18)
lemon	8.0 (43)	7.0 (41)	8.0 (44)
lime	6.0 (24)	1.0 (18)	1.5 (25)
marjoram	2.0 (21)	1.0 (30)	1.0 (20)
orange	5.0 (22)	1.0 (30)	6.0 (27)
palmarosa	2.0 (18)	3.5 (21)	0.5 (19)
peppermint	6.0 (20)	3.5 (23)	2.0 (23)
rosemary	1.0 (20)	0.5 (18)	0.0 (22)
sandalwood	2.0 (21)	6.5 (22)	1.5 (22)
spearmint	4.5 (18)	3.0 (18)	1.0 (19)
ylang ylang	2.0 (19)	5.5 (24)	0.0 (24)
**(2) linalool and its enantiometric isomers**			
linalool	5.5 (20)	5.0 (22)	1.0 (19)
( *RS*)-(±)-linalool	4.0 (18)	6.5 (21)	–
( *R*)-(−)-linalool	4.5 (23)	7.0 (24)	–
( *S*)-(+)-linalool	3.5 (26)	4.0 (23)	–

In addition, the value of total significance score, that is, the total sum of the statistical scores of each descriptor: 1.0 for the item with * (asterisk); 0.5 for that with ±; 0.0 for that unmarked, was calculated as 6.0 for peppermint, 4.5 for spearmint and 5.5 for linalool in relation to mental arithmetic, and 3.5 for peppermint, 3.0 for spearmint and 5.0 for linalool in relation to the auditory task. 

It should be noted that the changes of the spectra in Figure 1 were insignificant as a whole, because the obtained values of total significance score were all less than 10. On the basis of the sign test with *n* = 13 (as 13 pairs of impression descriptors were used in our sensory tests), the value should be required to be >10.

## 4. Discrimination between Enantiomers of Linalool in Terms of the Sensory Evaluation Spectrum as a Function of Behavioural Task

In this section, we describe our research on odour discrimination of enantiomers of linalool, quantified with the sensory evaluation spectrum and observed as a function of behavioural task [25,27].

Optically active linalools [(*R*)-(–)-, (*S*)-(+)- and (*RS*)-(±)-forms] were obtained by repeated flash column chromatography on silica gel (solvent: hexane/ethyl acetate, 9:1, v/v) of lavender oil, coriander oil and commercial linalool (see Sugawara *et al.*, [26] for details). For example, lavender oil (2.0 g) was subjected to flash chromatography on silica gel to yield linalool (439 mg), and its spectral (EI-MS, IR and ^1^H-NMR), chromatographic (analytical thin-layer chromatography; TLC, Merck 60 GF254 silica gel plate), and gas liquid chromatography (GLC; CP-cyclodextrin-β-236M-19) behaviour was compared with those of authentic specimens (kindly supplied by Dr. Y. Hiraga of Hiroshima University, Japan). The linalool was identified as (*R*)-(−)-linalool based on co-GLC analysis with an authentic (*R*)-form and was found to have a specific rotation of [α]_D_ = −15.1°, a 97.0% purity on GLC and a 21.95% total yield.

Using the same method, (*S*)-(+)-linalool was obtained from coriander oil, with a 32.9% total yield, a content of the (*S*)-form of 88.3% and (*R*)-form 11.7% on GLC, and [α]_D_ = +17.4°. Similarly, (*RS*)-(±)-linalool with a 74.5% total yield, a content of the (*S*)-form of 49.1% and (*R*)-form 50.9% on GLC, and [α]_D_ = 0° was re-purified from commercial linalool. 

**Figure 2:**
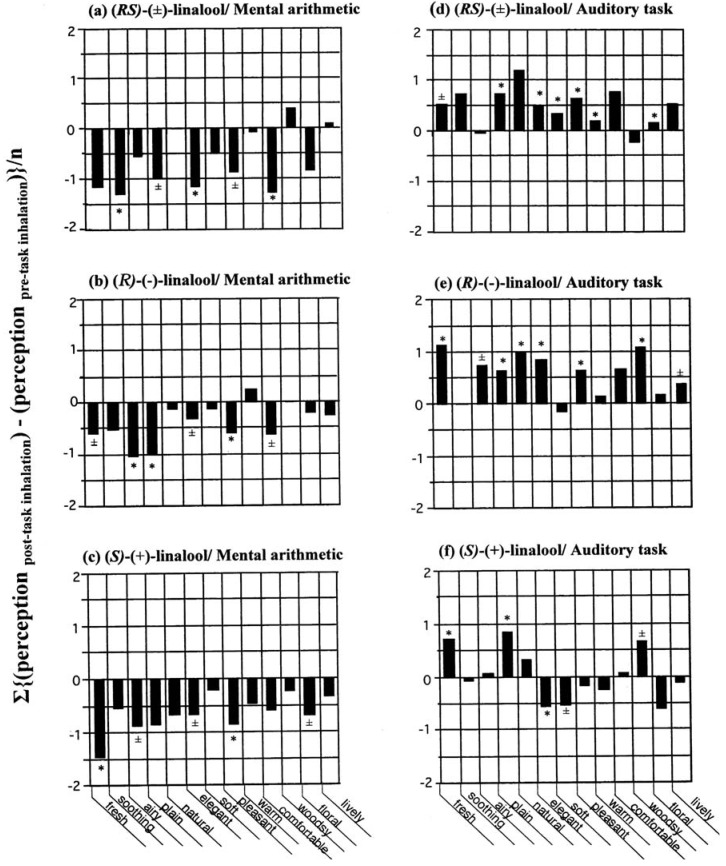
Sensory spectra of enantiomers of linalool [(*RS*)-(±)-, (*R*)-(−)- and (*S*)-(+)-forms] as a function of behavioural task. (**a**) (*RS*)-(±)-linalool, (**b**) (*R*)-(−)-linalool and (**c**) (*S*)-(+)-linalool when the subjects undertaking mental arithmetic; and (**d**) (*RS*)-(±)-linalool, (**e**) (*R*)-(−)-linalool and (**f**) (*S*)-(+)-linalool while undertaking the auditory task. Concentration of enantiomer was every 20 mg/mL (in diethyl phthalate), which was loaded and moistened in an inhalator (300 mL vol.) by applying 200 μL of each solution. Number of subjects was (**a**) 18, (**b**) 23, (**c**) 26, (**d**) 21, (**e**) 24 and (**f**) 23.

We used 20 mg/mL solutions of the enantiomers for sensory profiling because this concentration was rated as at least level 3 (“weak odour but the odour can be readily detected”) by the judges when they were presented with 50, 20, 10, 1 and 0.1 mg/mL solutions. The sensory spectra obtained using the optically active linalools in combination with behavioural tasks are summarized in Figure 2. Since (*RS*)-(±)-linalool was purified from commercial linalool and identified as a racemic mixture of the (*R*)-form (50.9%) and (*S*)-form (49.1%) with [α]_D_ = 0°, we used (*RS*)-(±)-linalool as a reference. The sensory evaluation spectra for purified linalool (Figure 2a,d) showed practically the same task-dependence of perception as the commercial linalool (Figure 1c,f). The total significance score of purified linalool was 4.0 for the mental arithmetic task (Figure 2a) and 6.5 for the auditory task (Figure 2d). This was in good agreement with the scores obtained using the commercial linalool, which were 5.5 with mental arithmetic (Figure 1c) and 5.0 with the auditory task (Figure 1f).

Figure 2 shows the sensory evaluation spectra from participants inhaling either (*R*)-(–)- or (*S*)-(+)-forms of linalool while undertaking the mental arithmetic or auditory tasks. (*RS*)-(±)-linalool was used as a reference. After mental arithmetic (Figure 2a–c, left hand side) the spectra showed negative values, indicating that the perceived odour quality of (*R*)-(–)- and (*S*)-(+)-linalools and the racemic mixture of the (*R*)- and (*S*)-forms was less favourable after the task. After the auditory task (Figure 2d–f, right hand side) the spectra showed mostly positive values. Thus, the spectra were completely reversed after the auditory task compared with the mental arithmetic task. Furthermore, a closer examination of the spectra obtained for the auditory task made it obvious that the sensory spectrum of (*R*)-(–)-linalool was identical to that of the reference (*RS*)-(±)-linalool, but not to that of (*S*)-(+)-linalool, for which half of the significant descriptors were positive and the other half negative.

None of the spectra shown in Figure 2 reached statistical significance as a whole, because the total significance scores were all ≤10 (Table 1). However, given these findings and the fact that Ohloff and Klein suggested (+)- and (–)-linalool are petitgrain-like and lavender-like, respectively [6], it is interesting to speculate that different enantiomers of linalool may evoke distinct odour perception in a task-dependent manner.

## 5. A Duplication of the Sensory Experiments

Here we describe the results of a series of experiments that duplicated our sensory profiling data [29]. In this experiment, the following dilutions were employed: 1/5 for lemon (as above-mentioned); 1/10 for cinnamon, cypress and orange; and 1/100 for marjoram.

In order to ascertain reproducibility and consistency of our results above, a duplication of sensory profiling was conducted for the following conditions: cypress and lemon *versus* the physical task; cinnamon *versus* mental arithmetic and the auditory task; marjoram and orange *versus* the auditory task; and lemon *versus* mental arithmetic and the auditory task. Two sets of mutually different panels were used in each run, with a total of 289 subjects completing the study.

**Figure 3:**
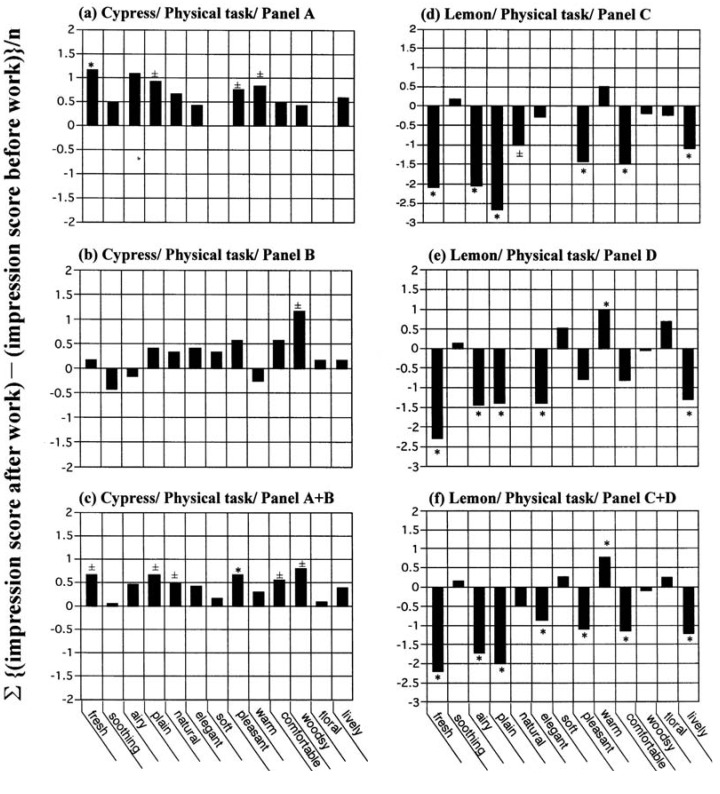
A duplication of sensory profiling conducted for cypress and lemon each *versus* physical task. In each column, a duplication of sensory profiling was conducted using two sets of mutually different panels, where no participant overlapped as panelist. The number of subjects was (**a**) 12 for Panel A, (**b**) 12 for Panel B and (**c**) 24 for Panel A+B as with cypress *versus* physical task; (**d**) 21 for Panel C, (**e**) 23 for Panel D and (**f**) 44 for Panel C+D as with lemon *versus* physical task. The total significance scores were 2.5 for (**a**), 0.5 for (**b**), 3.5 for (**c**), 6.5 for (**d**), 6.0 for (**e**) and 8.0 for (**f**).

Twelve subjects formed panel A and another 12 formed panel B, and both groups participated in the test of cypress *versus* physical task (Figure 3a–c). For the study of lemon *versus* physical task, 21 and 23 subjects formed panels C and D, respectively (Figure 3d–f). For each experimental run, we averaged the spectra from the two panels to form a combined spectrum. As shown in Figure 3, there was no discrepancy between the averaged spectrum (Figure 3c,f) and the individual spectra (Figure 3a,b,d,e). This was not only true for the shape of the spectra, but also for the total significance scores. The total significance scores were 2.5 for panel A, 0.5 for panel B, and 3.5 for panel A + B (cypress *versus* physical task); and 6.5 for panel C, 6.0 for panel D, and 8.0 for panel C + D (lemon *versus* physical task).

**Figure 4:**
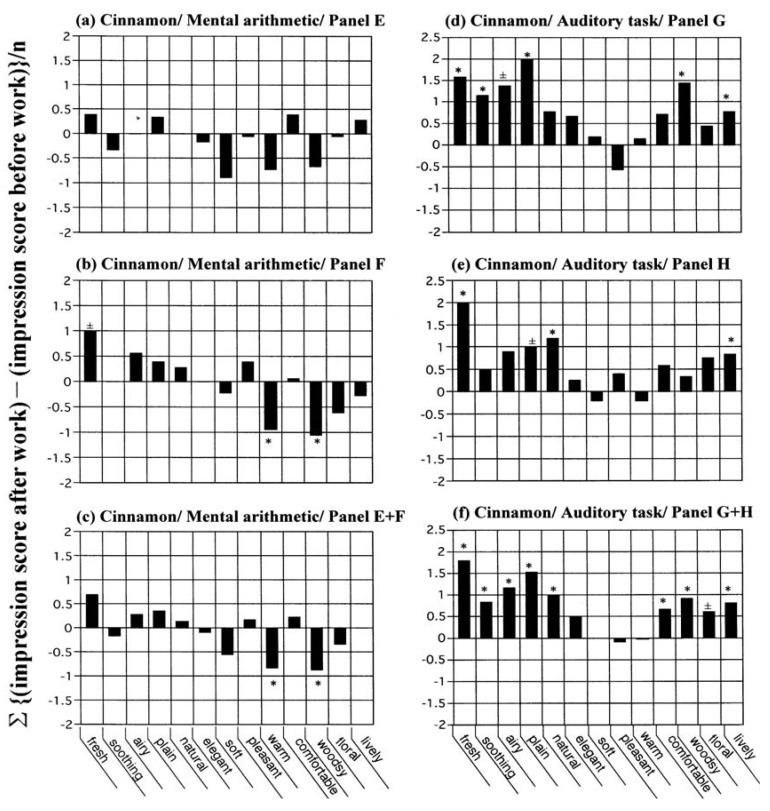
A duplication of sensory profiling conducted for cinnamon each *versus* mental arithmetic and the auditory task. All other conditions were identical with those in Figure 3. The number of subjects was (**a**) 18 for Panel E, (**b**) 18 for Panel F and (**c**) 36 for Panel E + F as with cinnamon *versus* mental arithmetic; (**d**) 21 for Panel G, (**e**) 20 for Panel H and (**f**) 41 for Panel G + H as with cinnamon *versus* the auditory task. The total significance scores were calculated as 0.0 for (**a**), 2.5 for (**b**), 2.0 for (**c**), 5.5 for (**d**), 3.5 for (**e**) and 8.5 for (**f**).

For the study of cinnamon *versus* mental arithmetic, 18 subjects formed panel E and 18 subjects formed panel F (Figure 4a–c). For the study of cinnamon *versus* the auditory task, another 21 and 20 subjects formed panels G and H, respectively (Figure 4d–f). Figure 4 shows that there was no discrepancy between the individual and pooled panels, in terms of shape or total significance scores. The total significance scores were 0.0 for panel E, 2.5 for panel F, and 2.0 for panel E + F (cinnamon *versus* mental arithmetic); and 5.5 for panel G, 3.5 for panel H, and 8.5 for panel G + H (cinnamon *versus* the auditory task). None of the spectra illustrated in Figure 3 and Figure 4 reached statistical significance as a whole, because the total significance scores were <10.

In the same fashion, another 15 and 15 subjects produced panels I and J, and participated in the study of marjoram *versus* the auditory task. Another 15 and 15 subjects formed panels K and L, and participated in the study of orange *versus* the auditory task. For the study of lemon *versus* mental arithmetic, 20 and 23 subjects formed panels M and N, respectively. For the study of lemon *versus* the auditory task, 19 and 22 subjects formed panels O and P, respectively. A detailed description of the outcome of these tests is not needed here. Apart from some discrepancies (lower repeatability and lower consistency) observed for lemon *versus* mental arithmetic and lemon *versus* the auditory task, we concluded that the findings from this series of duplicate sensory tests suggest a satisfactory to good agreement in terms of reproducibility and consistency of our sensory evaluation spectrum method.

## 6. Human Verbal and Non-Verbal Responses to Odours of Peppermint and Spearmint Essential Oils and Linalool in Relation to Different Behavioural Tasks

There were no sensory spectra with a total significance score of >10 among the 72 cases listed in Table 1, because of extraordinary deviation scatter of both pre- and post-task descriptor scores [24,25]. Therefore, we next used a multi-channel skin thermometer to monitor possible skin temperature changes and compare these with the sensory evaluation spectra, with the aim to examine the relationship between the psychological and physiological effects of odours. We will here describe findings from our first study that used peppermint and spearmint essential oils and linalool. These findings have been reported in the *Flavour and Fragrance Journal* [30] and the *International Journal of Essential Oil Therapeutics* [15].

Skin temperature measurements were conducted in a climatic chamber at 20 °C and 60% relative humidity. The procedure was first explained to the subjects, and they were then encouraged to relax and allowed to rest quietly for 5 min before the test began. None of the subjects were suffering from any chronic diseases, nasal congestion, or upper respiratory tract infection, and no one was taking any medication, remedies, or the contraceptive pill. To avoid potential confounding effects of hormonal profile and physiological status, female subjects did not participate in the study during menses. The ambient noise was kept at a low level (30–40 dBA) during the experiments.

Skin temperature was recorded using a thermometer (Anritsu AM-7052) equipped with a multi-channel data collector. Thin surface thermistors (2 × 10 mm) were used as sensors. They were attached with adhesive tape to the tips of all fingers of the left hand and to the palm at the base of the first finger of the left hand.

Various parts of the body have different temperatures and undergo circadian fluctuations. A pilot run was conducted with the thermistors affixed to the left cheek, left earlobe, forehead, tip of the left first finger, left palm (at the base of the first finger), and back of the left hand (at the base of the first finger). An odourless blank was used as a negative control. The skin temperature curve obtained from the tip of the left first finger and left palm showed a small but significant increase after odour inhalation, while practically no change was observed at the other measurement points [32]. Therefore, we developed a multi-channel thermometric technique by which skin temperature could be measured from the finger tips and palm of the left hand [28,30].

**Figure 5:**
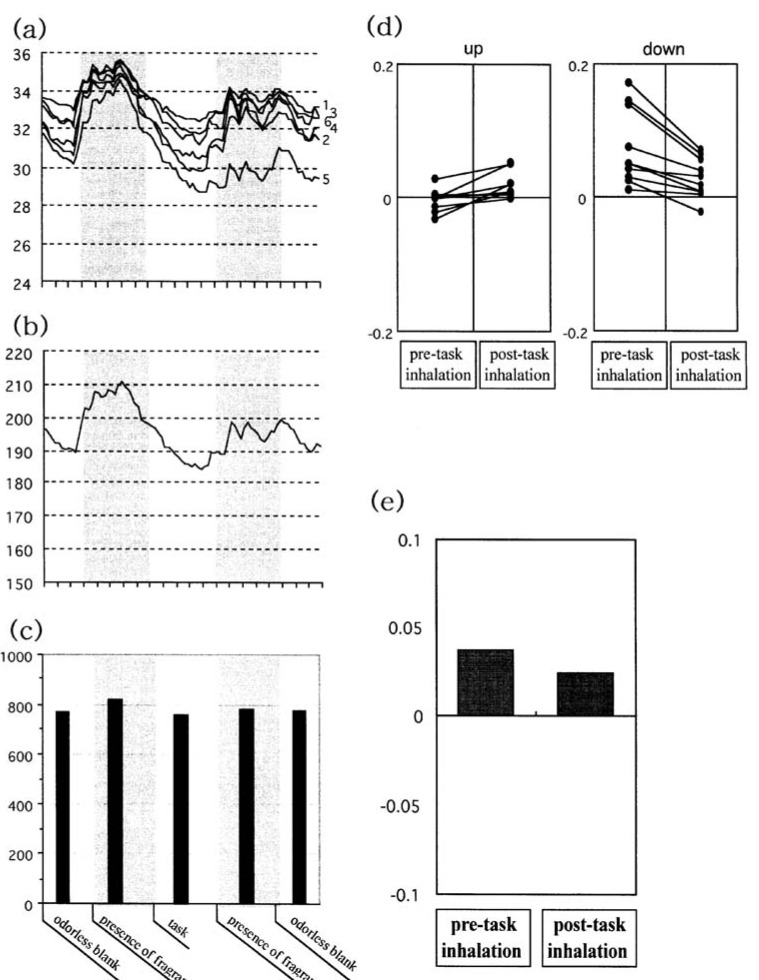
The observed skin temperature changes following inhalation of peppermint in association with the auditory task. Redrawn from Sugawara *et al.* [15]. The numbers assigned to the graph represent the sensor spots on the left hand: 1, the tip of the thumb; 2, the tip of the first finger; 3, the tip of the second finger; 4, the tip of the third finger; 5, the tip of the fourth finger, and 6, the palm. The number of subjects was 20.

All data were stored on a computer (Dell-OptiPlex Gn+EM) at a sampling rate of 15 s via an A/D converter connected to the multi-channel thermometer. This allowed summation of the data from each channel every 15 s so that a single temperature curve could be obtained for the six measurement points (Figure 5b). The experimental protocol was as follows: (1) inhalation of the odourless blank (total inhalation: 3 min; presence of the odourless blank: 30 s), (2) inhalation of essential oil before the task (total: 5 min, presence of the fragrance: 1 min), (3) 5 min of the task, (4) post-task inhalation of essential oil (total: 5 min; presence of the fragrance: 1 min), and (5) inhalation of the odourless blank (total: 3 min; presence of the odourless blank: 30 s).

For each experimental run, a temperature profile (bar chart) was constructed by the integration of temperature curves per minute for each section of the skin temperature measurement protocol (mean temperatures at one-minute intervals). We were then able to calculate the net skin temperature change between presentation of the odourless blank and the target fragrance: (T^MMA^_odour_ − T^MMA^_o_)/T^MMA^_o_, where T^MMA^_odour_ is the mean minute-based average temperature during odour presentation, and T^MMA^_o_ is the intensity during presentation of the odourless blank.

Figure 5 shows an example of skin temperature results from subjects who inhaled peppermint before and after the auditory task. As shown in Figure 5a (individual curves) and 5b (summation), the skin temperature curves showed a small but considerable change during odour presentation. However, there was considerable individual variation between trials in an experimental run. Therefore, for each trial, the minute-based mean average temperature was calculated for the different odour presentation periods (Figure 5c). On the basis of the minute-based temperature profile, the net change in skin temperature for each experimental run was calculated according to the formula described above. This method produced the results shown in Figure 5d,e. In Figure 5d, the cases showing an increase in skin temperature after the task are plotted in the left diagram, while those showing a decrease are represented in the right diagram. Figure 5e shows the overall mean values of the net temperature changes between pre- and post-task inhalations. Similar results were found for spearmint and linalool.

**Figure 6:**
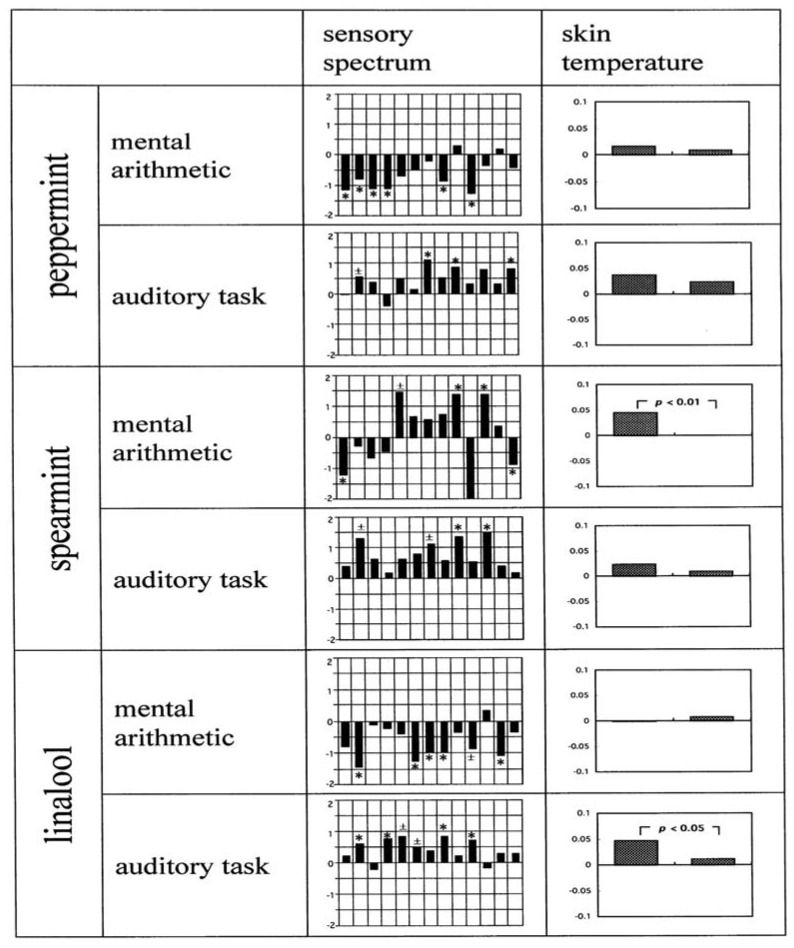
Summary of the verbal and non-verbal responses following inhalation of peppermint and spearmint essential oils and linalool in terms of the sensory evaluation spectrum and skin temperature changes as a function of behavioural task. Redrawn from Sugawara *et al.* [16]. Regarding sensory profiling study, the number of subjects was identical to that shown in Figure 1. As with skin temperature measurement study, the number of subjects was 18 for peppermint, 17 for spearmint and 20 for linalool in association with mental arithmetic; and 20 for peppermint, 18 for spearmint and 20 for linalool in relation to the auditory task.

The responses to inhalation of peppermint and spearmint essential oils and linalool are summarized in Figure 6, which illustrates the sensory evaluation spectrum and skin temperature changes as a function of behavioural tasks. A significant decrease in skin temperature between pre- and post-task was observed for spearmint after mental arithmetic (at *p* < 0.01): | *t*_0_ | = 4.153 ≥ *t*_d.f._ (d.f. = 16, *p* = 0.01) = 2.898. A decrease in temperature was also seen for linalool after the auditory task (at *p* < 0.05): | *t*_0_ | = 2.593 ≥ *t*_d.f._ (d.f. = 19, *p* = 0.05) = 2.086. Except for linalool *versus* mental arithmetic, where subjects experienced a small increase in temperature, a non-significant trend toward a decrease was seen for peppermint *versus* mental arithmetic and the auditory task, and for spearmint *versus* the auditory task.

## 7. Task-Dependence of Verbal and Non-Verbal Responses to Fragrances of Essential Oils of Lemon and Ylang Ylang

Based on results from the pilot study described in the preceding section, our most recent study concentrated on two tasks (mental arithmetic and auditory) and the following 10 essential oils: basil (*Ocimum basilicum*), bergamot (*Citrus bergamia*), cardamom (*Elettaria cardamomum*), cinnamon (*Cinnamomum zeylanicum*), juniper (*Juniperus communis*), lemon (*Citrus limon*), orange (*Citrus sinensis*), palmarosa (*Cymbopogon martini*), sandalwood (*Santalum album*) and ylang ylang (*Cananga odorata*). Among these, the effects of lemon and ylang ylang deserve special emphasis and are detailed below.

**Figure 7:**
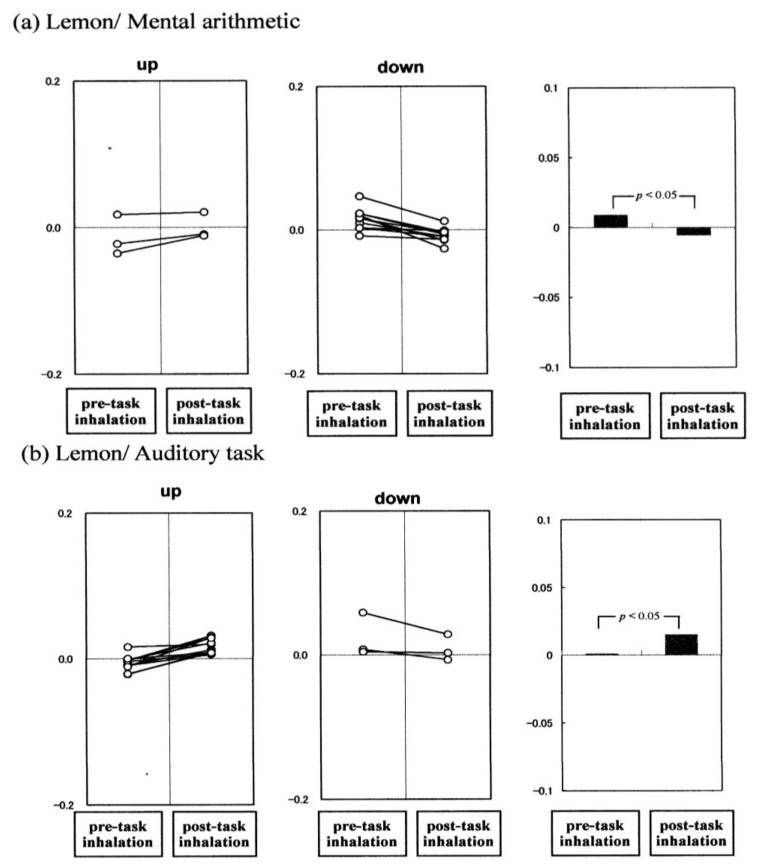
Skin temperature changes following inhalation of lemon as a function of behavioural task. The net intensity change in skin temperature (see text) was calculated between pre- and post-task inhalations with respect to presentation of the odourless blank and the target fragrance in each trial. In line segment graph, these are connected by a solid line for each subject in each experimental run and the cases showing upward skin temperature changes after the task are plotted on the left, while those with a downward tendency are represented in the middle panels. The summarized mean values of net intensity changes obtained from pre and post task inhalations are depicted respectively as bar graph on the right. (**a**) The number of subjects was 18 as for lemon/mental arithmetic; and (**b**) was 20 as with lemon/auditory task.

**Figure 8:**
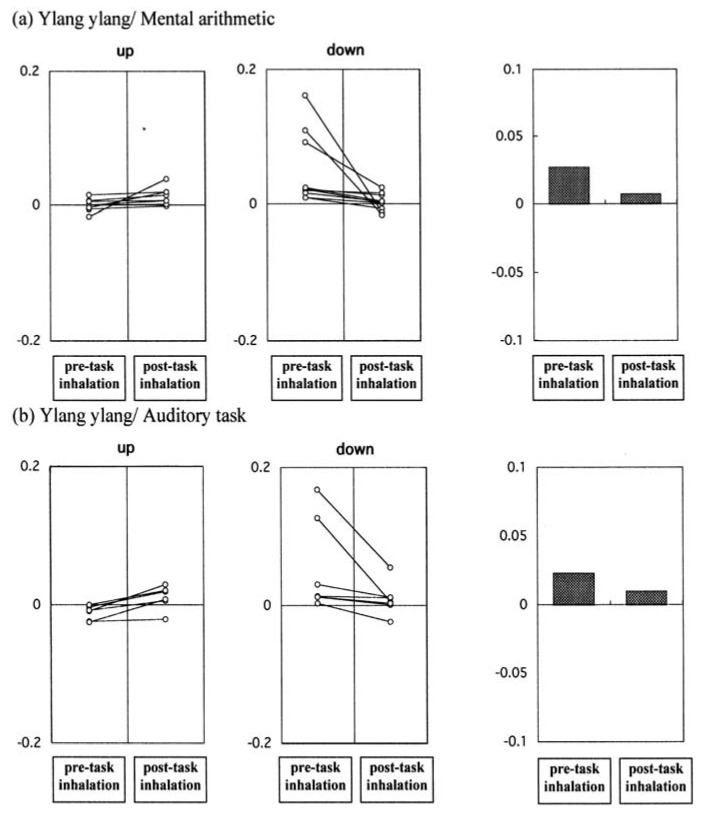
Skin temperature changes following inhalation of ylang ylang as a function of behavioural task. The circumstances are identical to those shown in Figure 5. (**a**) The number of subjects was 18 as for ylang ylang/mental arithmetic; and (**b**) was 20 as with ylang ylang/auditory task.

Figure 7 and Figure 8 show the skin temperature changes following inhalation of lemon and ylang ylang as a function of behavioural task. The methodology was identical to that used in the previous section as well as in Figure 5. The temperature response to lemon showed opposite signs for the two behavioural tasks: a significant decrease in skin temperature between pre- and post-task inhalations for mental arithmetic (at *p* < 0.05): | *t*_0_ | = 2.585 ≥ *t*_d.f._ (d.f. = 12, *p* = 0.05) = 2.179, and a significant increase for the auditory task (at *p* < 0.05): | *t*_0_ | = 2.655 ≥ *t*_d.f._ (d.f. = 13, *p* = 0.05) = 2.145 (Figure 7). For ylang ylang, a decrease was observed after both mental arithmetic and the auditory task, but this change did not reach statistical significance (Figure 8).

**Figure 9:**
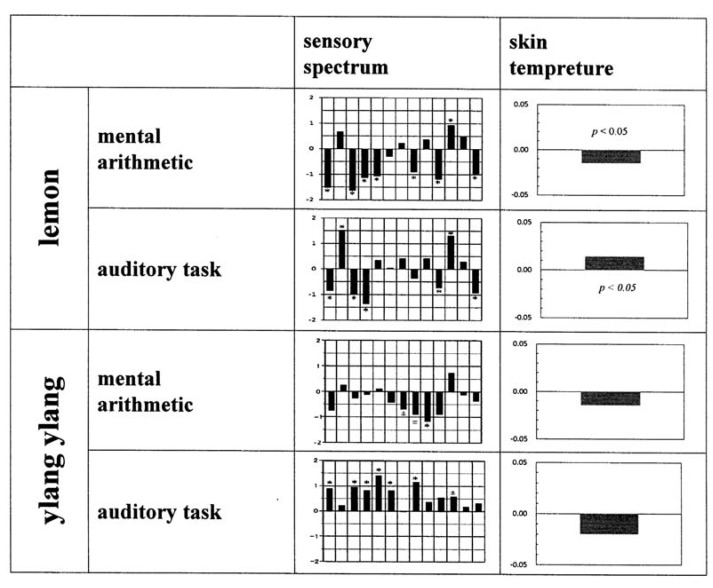
Summary of the verbal and non-verbal responses following inhalation of lemon and ylang ylang essential oils in terms of the sensory evaluation spectrum and skin temperature changes as a function of behavioural task. The number of subjects as for sensory profiling study was 43 for lemon/mental arithmetic, 41 for lemon/auditory task, 19 for ylang ylang/mental arithmetic and 24 for ylang ylang/auditory task. In regards to the number of subjects concerning skin temperature study, see Figure 7 and Figure 8.

Figure 9 represents a summary of the effects of inhalation of lemon and ylang ylang on the sensory evaluation spectrum and skin temperature. The sensory spectra obtained for lemon did not reveal any task-dependence; both tasks resulted in a negative correlation between the odour and the behavioural task. However, lemon had a pronounced task-dependent dual effect on skin temperature: a significant decrease in temperature in subjects undertaking mental arithmetic and a significant increase in subjects undertaking the auditory task.

For ylang ylang, a dual task-related effect was instead seen in the sensory evaluation spectra. As shown in Figure 9, administration of ylang ylang resulted in a negative change in perception when subjects were undertaking mental arithmetic and a positive change when subjects were undertaking the auditory task. However, a decrease in skin temperature occurred in both tasks, although these changes did not reach statistical significance.

## 8. Discussion

Sensory analysis comprises a variety of tools and tests that can be used for subjective or objective evaluation of sensory properties. Our sensory test can be categorized as descriptive sensory analysis. Descriptive sensory analysis consists of training a group of individuals (generally 6 to 12) to identify and quantify specific sensory attributes [33,34,35,36]. In contrast to this, our research has used untrained individuals as panellists.

If untrained individuals are used as panellists, factors such as interest in sensory target, sensitivity to stimuli, susceptibility to fatigue, and so on vary between panellists. This was obvious from our questionnaire, in which the standard deviation of responses was remarkably large compared with the smaller value of the mean for the 13 descriptors, as described elsewhere [16,24,25]. Regardless of this, Figure 1, Figure 2, Figure 3, Figure 4 and Figure 5 demonstrate that the sensory spectra seem to represent several aspects of odour perception in participants (Figure 1, Figure 2, Figure 3 and Figure 4) and show satisfactory to good reproducibility (Figure 3 and Figure 4).

In this context, it is interesting to consider another of our sensory studies, in which sensory tests were carried out after placing a TiO_2_-type deodorizer in the participants’ own home refrigerators. The results were used as indices of the untrained participants’ perception of the smell within the refrigerator [16]. To ascertain the validity of this approach, a repeatability test was conducted to compare two studies that were undertaken in 2003 and 2004 in the same fashion. No participant was a panellist in both studies. In each experimental run, the questionnaire (composed of 13 contrasting adjective pairs) was administered three times: once immediately before placing the deodorant in the refrigerator, once after keeping it in the refrigerator for a week, and one week after removing it from the refrigerator. The scores obtained from the second questionnaire were subtracted from those obtained from the first (the second minus the first), and the scores from the third questionnaire were subtracted from the scores from the second (the third minus the second). Thus, for each run, two functional sensory spectra were obtained: one representing the change in perception after installation of the deodorant, and one representing the change after its removal. The first spectrum showed a positive effect on the perception of smell in the refrigerator, consistent with a removal of odours by the deodorizer. The second spectrum showed a negative effect in perception, indicating that the odors were intensified upon removal of the deodorizer. Even though the standard deviation was large compared with the mean, both functional spectra were significant (*p* < 0.05) as a whole, as the total significance scores were > 10.

These results differ a great deal from the present research, where no spectrum reached statistical significance (Table 1). Hence, we commenced a multi-channel skin thermometer measurement study to detect possible physiological changes that might complement the sensory evaluation spectra. Emotional excitement or apprehension is known to induce a slight increase in skin temperature [37,38,39,40,41]. As the perception of and response to odours are intimately related to both emotional expression and genesis of emotion, skin temperature changes can serve as an effective index for the study of the relationship between odour and its emotional and physiological effects in humans.

Although changes in skin temperature due to emotional stress have been demonstrated by several authors, the mechanisms by which odorants induce such changes are unknown. Based on findings that blood vessels are supplied only with vasoconstrictor efferents, it has been suggested that emotional stress leads to cutaneous vasoconstriction, thus lowering skin temperature [42,43,44,45,46,47]. The participation of sympathetic nerve-mediated vasodilation has also been reported [48,49,50], although no known vasodilator nerve fibres are connected to the cutaneous vessels in humans [51]. Elam and Wallin observed that mental stress caused vasoconstriction in warm subjects and vasodilation in cold subjects [38], suggesting that a thermoregulatory mechanism might exert a powerful modulatory effect on different cutaneous vasomotor reactions [38,52]. In addition, there are short channels in the fingers, palms and earlobes of humans that connect arterioles to venules, bypassing the capillaries. These arteriovenous anastomoses, or shunts, have thick muscular walls and are abundantly innervated, presumably by vasoconstrictor nerve fibres. The skin temperature also depends on the state of the capillaries and venules. In cold skin the arterioles are constricted and capillaries are dilated, while in warm skin both are dilated. Sweat production is another important factor in skin temperature regulation, and it can be affected by emotion through input from the sudomotor nerve fibres [53,54,55,56].

Regardless of the complexity of the factors that influence cutaneous vascular responses with respect to skin temperature changes, our findings showed that the essential oils could be roughly classified into two groups: those that caused an increase in fingertip skin temperature and those that caused a decrease (Figure 5, Figure 6, Figure 7, Figure 8 and Figure 9). If feelings of excitement or apprehension induce a slight increase in skin temperature [37,38,39,40,41], it is reasonable to assume that essential oils can be regarded as having distressing/agitating properties if they cause an increase in skin temperature, and relaxing/sedating properties if they cause a temperature decrease.

In our studies, we hypothesized that the combination of physiological and psychological measures of odour perception during different behaviours might be highly informative, not only for studying the potency of essential oils, but also for investigating olfactory discrimination and odour responses in humans. As shown in Figure 6 and Figure 9, the individual spectra reflect subtle aspects of olfactory discrimination, and when the perception of odorants is tested under different behavioural conditions they also illustrate finer nuances of psychophysiological expressions. Specifically, both behavioural tasks in combination with inhalation of ylang ylang were accompanied by relaxation/sedation (skin temperature decrease), but showed contrasting sensory evaluation spectra (Figure 9).

An intriguing observation was made by Hongratanaworakit and Buchbauer in 2004 and 2006 [17,57]. In their first study [17], the authors demonstrated that the effect of inhalation of ylang ylang could be characterized as harmonizing rather than relaxing/sedating. The authors found that inhalation of ylang ylang led to a decrease in blood pressure and pulse rate but also increased subjective attention and alertness. In other words, the concept of “harmonization” is consistent with otherwise contradictory psychophysiological outcomes. In their next report [57], the same authors reported that transdermal absorption of ylang ylang caused a decrease in blood pressure and an increase of subjective calmness and relaxation compared with the control experiment. Based on these findings, the authors suggested that ylang ylang has a relaxing/sedating effect when administered via transdermal absorption.

**Figure 10:**
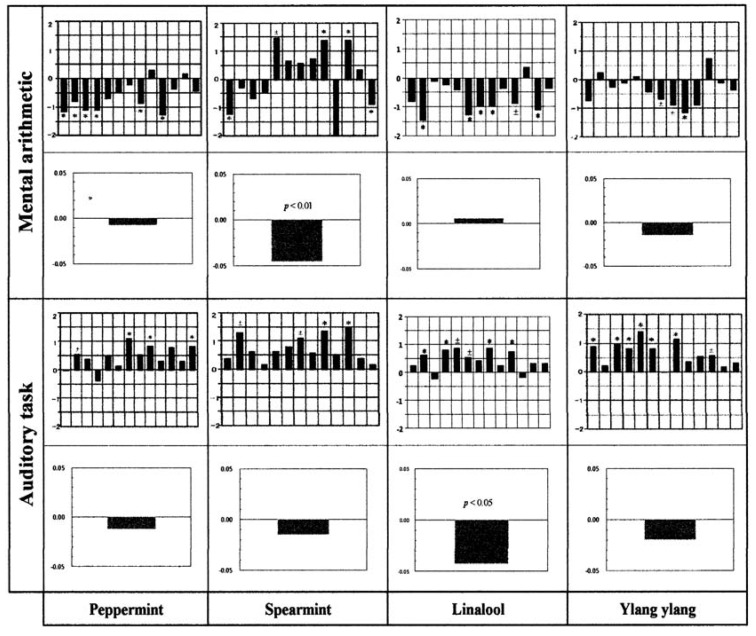
Essential oils showing “harmonization” in terms of the sensory spectra and skin temperature changes as a function of behavioural task. The conception of “harmonization” was introduced by Hongratanaworakit and Buchbauer as for ylang ylang [17]. From the view of this conception, we made attempts to search such an essential oil on the basis of our obtained task-dependent sensory spectra and fingertip skin temperature changes. With the ylang-ylang’s sensory spectra and net intensity fingertip skin temperature changes as a reference, two essential oils (peppermint and spearmint) and one monoterpenoid (linalool) were chosen.

On inspection of our findings from this view, the effect of ylang ylang seemed to be relaxing/sedating based on physiological and psychological measures when combined with the auditory task (Figure 9). However, the effect of ylang ylang was in good agreement with the concept of “harmonization” when mental arithmetic was used (Figure 9). Here, we attempt to expand the definition of harmonization to include the conditions in our study. This could allow understanding of the finer nuances of the contradictory changes observed for the two tasks. Using the graphs obtained for ylang ylang for the two tasks as a reference, we identified other odorants with similar properties (Figure 10). We found that peppermint and spearmint essential oils and linalool could be categorized as causing “harmonization” in terms of the task-dependent sensory spectra and skin temperature changes. A minor exception was seen with linalool *versus* mental arithmetic, where subjects experienced a small increase in fingertip skin temperature (within the measurement error) rather than a decrease. (*R*)-(–)-linalool in association with the auditory task was found to produce positive values of the sensory evaluation spectrum, and this effect was quite similar to that of (*RS*)-(±)-linalool but contrasted with that of (*S*)-(+)-linalool. In contrast, (*R*)-(–)-linalool in association with mental arithmetic produced an unfavourable change in perceived odour quality, and this feature closely resembled the effects of (*S*)-(+)-linalool and (*RS*)-(±)-linalool. Taken together with the previous report that the aromas of (+)- and (–)-linalool are petitgrain-like and lavender-like, respectively [6], our findings suggest that the enantiomers of linalool are significantly different odorants and also that the perception of them is task-dependent.

These findings are consistent with the views of Lorig and Schwartz [58], who pointed out that odours act as neurophysiological stimuli even when subjects do not report hedonic effects or intensity differences between odours. Because of these salient effects of odours and their ability to modify limbic system activity, aromas may have an application in the alteration of mood states in humans.

It is worthwhile mentioning the current state of knowledge of olfaction [7,8,9,10,11]. Approximately 1,000 different odorant receptors are present in hair-like cilia of olfactory neurons in the olfactory epithelium, where the initial detection of odours takes place. The genes encoding odorant receptors is considered to be up to 1% of the mammalian genome, making this the largest gene family thus far identified. Hence, there is a vast number and a great variety of odorant receptors at the entrance of the olfactory system, enabling discrimination of a large range of odours and subtle differences in the odorant molecules. In our studies, the fragrance given to the subjects was the same in different experimental runs, so we expect that a similar set of odorant receptors was activated in the different behavioural tasks. Even taking into account the great variety of odorant receptors, our studies indicate that the observed task-dependence of odour reactions reflects the nature of the odorants. A great deal is already known about the features of the olfactory epithelium [7,8,9,10,11,59,60], but very little is known about processes beyond that. Further work is needed to characterize the mechanisms of odour discrimination beyond the very first stages of olfactory processing.

Finally, it is possible that diversity in higher-order olfactory processing that cause the observed task-dependence may contribute to resolving several controversies often discussed in aroma research. We next discuss some disagreements about the psychophysiological effects of essential oils. Support for the hypothesis that lemon fragrance causes distress/agitation is provided by the observation that lemon aroma caused an increase of heart rate [61,62], whereas arguments against it were seen in a study by Manley [63], in which administration of lemon aroma through the air conditioning system resulted in a decrease of contingent negative variation (CNV) as well as a decrease of key entry errors of video display terminal (VDT) operators. Manley [63] also reported that ylang ylang aroma appeared to possess a stimulating effect, as it caused an increase of the CNV magnitude. Hongratanaworakit and Buchbauer [17,57] demonstrated that its effect can be characterized as relaxation/sedation. For peppermint, arguments in support of the supposition that it is stimulating came from observations that peppermint aroma caused an increase of electroencephalography (EEG) speed and heart rate during sleep [64], and increase of the CNV magnitude [63,65], a decrease of theta activity [66], and EEG and behavioural arousal during stage 1 sleep [67]. However, arguments against this supposition have emerged from the following: peppermint aroma produced significant increases of gross speed, net speed and accuracy in a typing task [68]; more NREM sleep, less REM sleep and more slow-wave sleep [69]; and increased alertness, decreased temporal demand and decreased frustration during simulated driving [70]. Despite these opposing views, the disagreements may in part be reflections of the diversity of higher-order olfactory processing. However, we will not achieve consensus until we have deeper insights into these processes. The key to solve these matters may be further studies of these phenomena as a function of external conditions such as behavioural tasks.

## 9. Conclusions

(1) This article is an overview of our research results over the past decade, which suggest that our tests of task-related sensory perception and skin temperature changes are useful for shedding more light on the effects of essential oils and on the finer nuances of odour discrimination and psychophysiological responses to odours in humans.

(2) Inhalation of (*R*)-(–)-linalool or (*RS*)-(±)-linalool in association with the auditory task produced a positive impression of odour quality, whereas this effect was not seen for (*S*)-(+)-linalool. In contrast, administration of (*R*)-(–)-linalool, (*S*)-(+)-linalool and (*RS*)-(±)-linalool in association with mental arithmetic all produced a negative impression of odour quality. This indicates that the enantiomers of linalool have different smells and that the perception of them is task-dependent.

(3) We also studied skin temperature changes as an index of mood changes during odour presentation. In our first experiments, lemon, peppermint, spearmint and ylang ylang essential oils as well as linalool were studied.

(4) Skin temperature measurements indicated that inhalation of ylang ylang was relaxing/sedating for both the auditory and mental arithmetic tasks. In contrast, the sensory spectra showed that mental arithmetic was associated with an unfavourable impression of the odour, whereas the auditory task was associated with a favourable impression.

(5) Our results were consistent with a previous suggestion that ylang ylang is “harmonizing” rather than relaxing/sedating [17]. When we used the obtained sensory perception profile of ylang ylang as a reference to evaluate the other tested odorants, we found that peppermint, spearmint and linalool could also be classified as “harmonizing”.

(6) Lemon aroma caused a task-dependent dual effect on skin temperature, with a decrease after mental arithmetic and an increase after the auditory task. However, the sensory evaluation spectra indicated that both tasks were associated with a negative change in the perceived odour quality of lemon.

(7) These findings remind us that odours act as neurophysiological stimuli and cause different perceptions, which in turn lead to diverse odour reactions that also depend on internal and external conditions of the subjects, as stated by Lorig and Schwartz [58].

(8) Odorant receptors are encoded by the largest gene family thus far identified, making it a very diverse group of receptors. In our studies, however, the fragrance administered to the subjects was well controlled and should activate the same set of receptors regardless of behavioural task. Hence, the observed task-dependence of odour perception and physiological responses likely originates from higher-order processes in the brain.

(9) A great deal is known about the initial stages of olfactory processing, but much less is known about processes beyond the olfactory bulb. Our research over the past decade indicates that further work is needed to characterize the mechanisms of odour discrimination beyond the nasal epithelium. Such information may provide answers to the long-standing questions of how we can identify so many odorants, why odours cause such strong emotions, how the variety of behavioural responses to odours are mediated, and to what extent our behaviour and mood are controlled by the perception of odours.
